# Mechanism Investigation of Rifampicin-Induced Liver Injury Using Comparative Toxicoproteomics in Mice

**DOI:** 10.3390/ijms18071417

**Published:** 2017-07-02

**Authors:** Ju-Hyun Kim, Woong Shik Nam, Sun Joo Kim, Oh Kwang Kwon, Eun Ji Seung, Jung Jae Jo, Riya Shresha, Tae Hee Lee, Tae Won Jeon, Sung Hwan Ki, Hye Suk Lee, Sangkyu Lee

**Affiliations:** 1 BK21 PLUS Team for Creative Leader Program for Pharmacomics-Based Future Pharmacy and Integrated Research Institute of Pharmaceutical Sciences, College of Pharmacy, The Catholic University of Korea, Bucheon 14662, Korea; jhyunkim@catholic.ac.kr; 2 BK21 Plus KNU Multi-Omics Based Creative Drug Research Team, College of Pharmacy, Research Institute of Pharmaceutical Sciences, Kyungpook National University, Daegu 41566, Korea; namhero@naver.com (W.S.N.); tjswn712@nate.com (S.J.K.); kok0100@nate.com (O.K.K.); ssungeunji3@gmail.com (E.J.S.); cho2j90@naver.com (J.J.J.); riya.shrestha07@gmail.com (R.S.); 3 Toxicological Research Center, Hoseo University, Asan 31499, Korea; thlee@hoseo.edu (T.H.L.); twjeon@hoseo.edu (T.W.J.); 4 College of Pharmacy, Chosun University, Gwangju 61452, Korea; shki@chosun.ac.kr

**Keywords:** rifampicin, drug-induced liver injury, anti-tuberculosis, proteomics

## Abstract

Tuberculosis is one of the top causes of death among curable infectious diseases; it is an airborne infectious disease that killed 1.1 million people worldwide in 2010. Anti-tuberculosis drug-induced liver injury is the primary cause of drug-induced liver injury (DILI). Rifampicin is one of the most common anti-tuberculosis therapies and has well-known hepatotoxicity. To understand the mechanism of rifampicin-induced liver injury, we performed a global proteomic analysis of liver proteins by LC-MS/MS in a mouse model after the oral administration of 177 and 442.5 mg/kg rifampicin (LD_10_ and LD_25_) for 14 days. Based on the biochemical parameters in the plasma after rifampicin treatment, the hepatotoxic effect of rifampicin in the mouse liver was defined as a mixed liver injury. In the present study, we identified 1101 proteins and quantified 1038 proteins. A total of 29 and 40 proteins were up-regulated and 27 and 118 proteins were down-regulated in response to 177 and 442.5 mg/kg rifampicin, respectively. Furthermore, we performed Gene Ontology (GO) and Kyoto Encyclopedia of Genes and Genomes (KEGG) pathway enrichment analyses to characterize the mechanism of rifampicin-induced hepatotoxicity. In the molecular function category, glutathione transferase activity was up-regulated and proteins related to arachidonic acid metabolism were down-regulated. In the KEGG pathway enrichment-based clustering analysis, the peroxisome proliferator-activated receptor-γ (PPARγ) signaling pathway, cytochrome P450, glutathione metabolism, chemical carcinogenesis, and related proteins increased dose-dependently in rifampicin-treated livers. Taken together, this study showed in-depth molecular mechanism of rifampicin-induced liver injury by comparative toxicoproteomics approach.

## 1. Introduction

The liver is a very important organ in drug metabolism; it lies between absorption and systematic circulation in terms of its function and it is the primary location of metabolism and the discharge of external substances. Owing to these properties, the liver is also considered the main target of drug toxicity. Drug-induced liver injury (DILI) is the most common side effect leading to the failure of new drug candidates or withdrawal from the market and is a major clinical problem [1]. Currently, over 1000 drugs are known to cause DILI, and the list is continuing to grow [2]. Drugs that most frequently cause DILI can be found in epidemiological data. According to an epidemiological study in China in 2013, the leading cause of DILI was tuberculosis drugs [3]. In Spain in 2005 and in the US in 2008, amoxicillin and clavulanate were classified as the major causes of DILI, and anti-tubercular drugs were ranked second and third, respectively [4,5].

Tuberculosis is one of top causes of death among curable infectious diseases. According to the World Health Organization (WHO) data published in 2016, 9.6 million people were suffering from tuberculosis and 1.5 million died from it in 2014 [6]. In accordance with the increase of human immunodeficiency virus (HIV) patients, an increase in the incidence of tuberculosis became a major problem worldwide [7]. There are four main tuberculosis treatment agents, i.e., isoniazid, rifampicin (RIF), pyrazinamide, and ethambutol. The typical regimen in clinical practice for adult respiratory tuberculosis includes isoniazid, RIF, and pyrazinamide for two months, followed by isoniazid and RIF for four additional months [8]. However, anti-tuberculosis drugs, such as isoniazid, RIF, and pyrazinamide, cause hepatotoxicity frequently [9].

Currently, RIF is one of the drugs of choice for tuberculosis, and it induced to hepatotoxicity [10]. RIF is known to cause hepatocellular dysfunction in the early stage of administration, but this symptom disappears after administration is stopped [11]. In addition, RIF affects bilirubin excretion by causing temporary hyperbilirubinemia [12]. Since it causes liver injury in the form of cholestasis, it is also related to distinctive hepatic lesions resulting from hepatocellular changes accompanied by centrilobular necrosis [13].

Since tuberculosis is generally treated with multiple drugs, except when isoniazid monotherapy is used for latent tuberculosis infection, there is limited information regarding the pathogenesis of toxicity for each tuberculosis drug. Although hepatotoxicity is one of main side effects of tuberculosis treatment, especially for RIF, the mechanism of RIF-induced liver injury has not been clearly determined. In the present study, to investigate the mechanism of RIF-induced liver injury, we performed a global proteomic analysis of RIF-treated livers using nano-flow liquid chromatography with tandem mass spectrometry (nLC-MS/MS) in a mouse model.

## 2. Results

### 2.1. Rifampicin-Induced Hepatotoxicity

**Figure 1:**
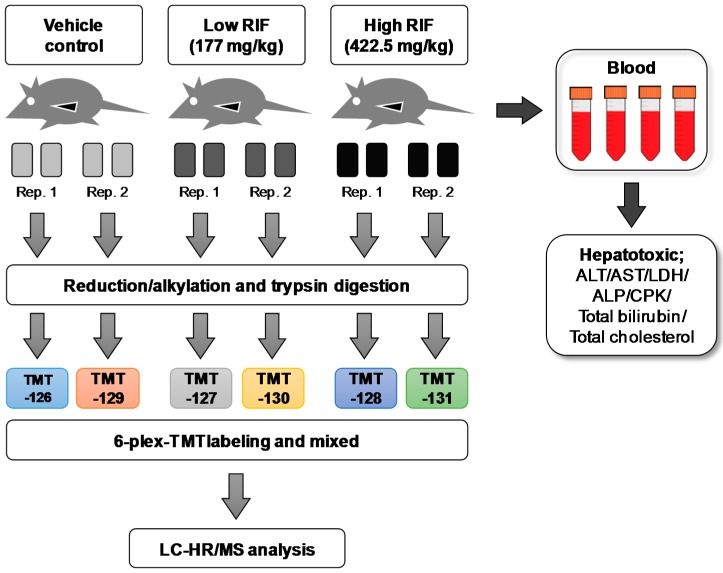
Experimental scheme for proteomic profiling of rifampicin-induced liver injury in mice. Four male ICR mice in each group were orally administered with 177 and 442.5 mg/kg rifampicin in 5% (*v*/*v*) dimethyl sulfoxide and 25% (*v*/*v*) polyethylene glycol (PEG) in water for 14 consecutive days. All animals were subjected to necropsy in 24 h after treatment. RIF: rifampicin; Rep.: replicate; TMT: tandem mass tag; LC-HR/MS: liquid chromatography-high resolution mass spectrometry; ALT: alanine aminotransferase; AST: aspartate transaminase; LDH: lactate dehydrogenase; ALP: alkaline phosphatase; CPK: creatine phosphokinase.

To investigate its hepatotoxic mechanism at the molecular level, RIF was orally administered to mice at doses of 177 and 442.5 mg/kg (LD_10_ and LD_25_) for 14 days (Figure 1). To examine the general toxic effect of RIF, changes in body and liver weights were determined during the 14-day experimental period. As shown in Table 1, body weight was not affected by RIF administration. However, liver weights increased significantly by 1.7- and 2.7-fold compared with those of the vehicle control groups after treatment for 14 days with 177 and 442.5 mg/kg RIF, respectively. The liver weights relative to body weights also increased significantly by 1.7- and 2.2-fold for 177 and 442.5 mg/kg RIF, respectively. The dose-dependent increases of liver weights with both 177 and 442.5 mg/kg RIF indicate the initiation of a liver abnormality like fat accumulation.

The hepatotoxicity induced by RIF was investigated based on blood parameters after oral administration for 14 days (Table 2 and Table S1). In response to 177 and 442.5 mg/kg RIF, alanine aminotransferase (ALT) activity, an indicating parameter of liver specific damage, in mice increased significantly by 2.1- and 3.4-fold compared to that of the vehicle group, respectively (Table 2). The other parameters for detecting liver damage, the activity levels of aspartate transaminase (AST) and lactate dehydrogenase (LDH), increased significantly by 2.9- and 3.1-fold, respectively, for 442.5 mg/kg RIF (Table 2 and Table S1). However, the activity of alkaline phosphatase (ALP), another parameter of liver damage derived from abnormal biliary excretion, did not increase after RIF administration for 14 days. Additionally, RIF resulted in hyperbilirubinemia; total bilirubin in the blood increased by 4.5- and 9-fold for doses of 177 and 442.5 mg/kg, respectively, while the triglyceride level with 442.5 mg/kg RIF was a half of that of the vehicle group. Total cholesterol increased significantly by 2.3- and 3.7-fold for 177 and 442.5 mg/kg RIF, respectively. Low-density lipoprotein (LDL) cholesterol increased by 4.4- and 5.2-fold, but HDL-C did not increase (Table S1). Following on Roussel Uclaf Causality Assessment Method (RUCAM) classification, the *R* value (*R* = ALT/ALP) at 177 and 442.5 mg/kg were calculated as 2.1 and 3.4, respectively. We defined the hepatotoxic mechanism of RIF as mixed liver injury because the R values were estimated at the range of 2 < *R* < 5 [14]. These patterns of parameter changes after RIF administration in mice were consistent with those of a previous study [15].

**Figure 2:**
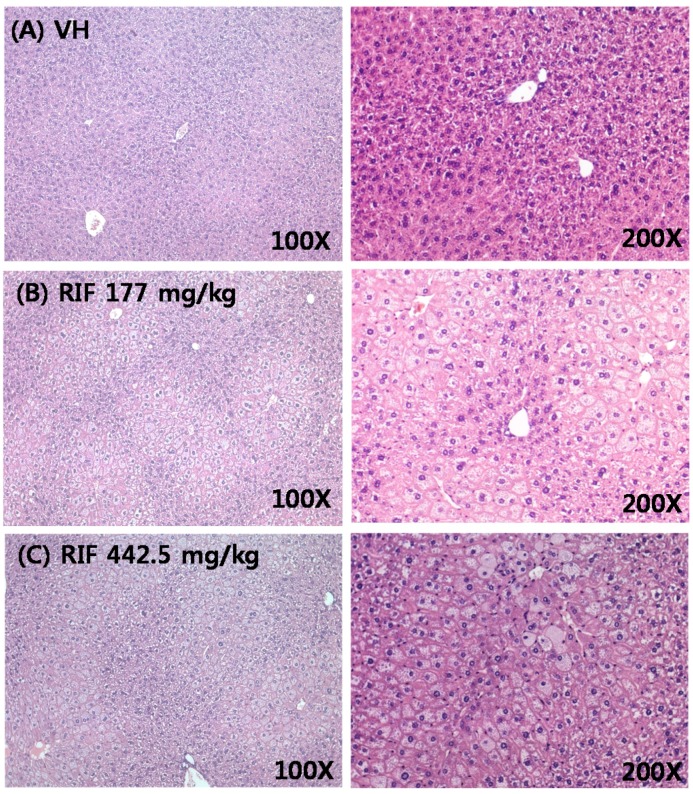
Histopathology of liver tissue in male ICR mice. The liver tissue of vehicle (**A**), rifampicin-treated at 177 mg/kg (**B**); and rifampicin-treated at 442.5 mg/kg (**C**) for 14 days at 100× and 200× magnification.

The largest and the second largest lobes were cut from the mouse livers and slides were prepared. They were H&E-stained and observed at 100× and 200× magnification (Figure 2). We observed extreme hepatocyte hypertrophy characterized by a notable increase in cell size accompanied by binucleate hepatocytes with enlarged hepatocyte nuclei.

RIF is an agonist of pregnane X receptor (PXR), and PXR protein overexpression and activation by RIF increases the metabolism of cytochrome P450 (CYP) 3A4 substrates [16]. Accordingly, mouse livers were homogenized to produce the S9 fraction and the activity levels of CYP1A, 2B, 2C, 2D, and 3A were determined by a cocktail probe assay [17]. As shown in Figure S1A, CYP2C-mediated omeprazole 5-hydroxylation increased significantly by 3.4-fold compared with that of the vehicle control group after 14 days of treatment with 442.5 mg/kg RIF. CYP3A-catalyzed midazolam hydroxylation increased slightly after RIF treatment in a dose-dependent manner.

### 2.2. Quantitative Proteomic Analysis of RIF-Administered Liver Proteins

To investigate the mechanism of toxicity in mouse livers after the administration of 177 and 442.5 mg/kg RIF, liver proteins were analyzed by global proteomics coupled with a chemical tagging method (Figure 1). The pooled protein homogenates were trypsin-digested, and the tryptic peptides were subjected to 3-plex isobaric TMT tag analyses by 2D-LC using a hybrid quadrupole-orbitrap mass spectrometer. All MS/MS spectra were analyzed using MaxQuant software, allowing a maximum FDR of 1% for the proteins and peptides. To obtain a high-quality protein list, we excluded contamination and results with less than one unique peptide/protein from the total protein list. 

In total, 1101 proteins were identified, among which 1038 proteins were quantified. All data consisted of technical duplicates. Proteins were divided into two categories based on relative levels; those with a quantitative ratio of greater than 1.5 were considered up-regulated, while those with a quantitative ratio of less than 0.667 were considered down-regulated (Table S2).

**Figure 3:**
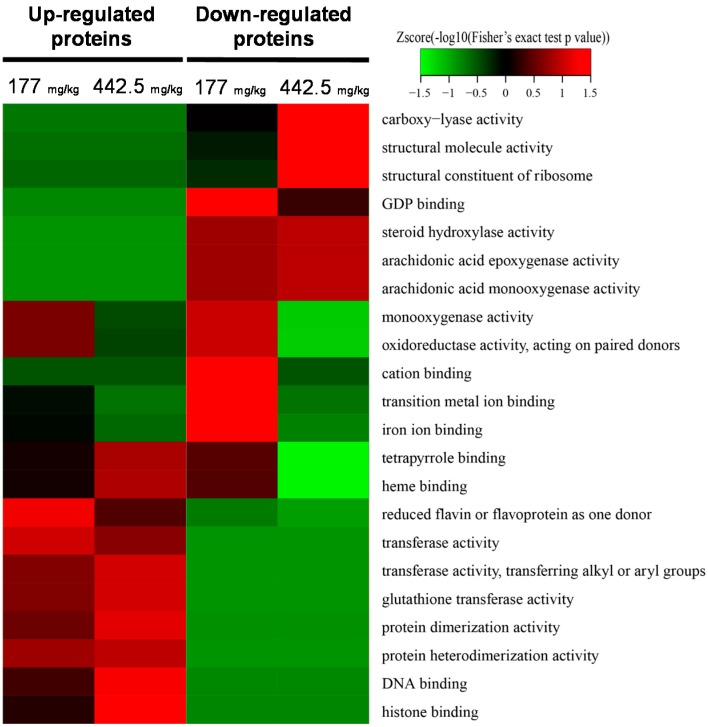
Gene ontology functional classification of quantified proteins based on molecular function. The heat map was generated using the two-tailed Fisher’s exact test to test the enrichment of each differentially expressed protein against all identified proteins. *p*-value < 0.05.

**Figure 4:**
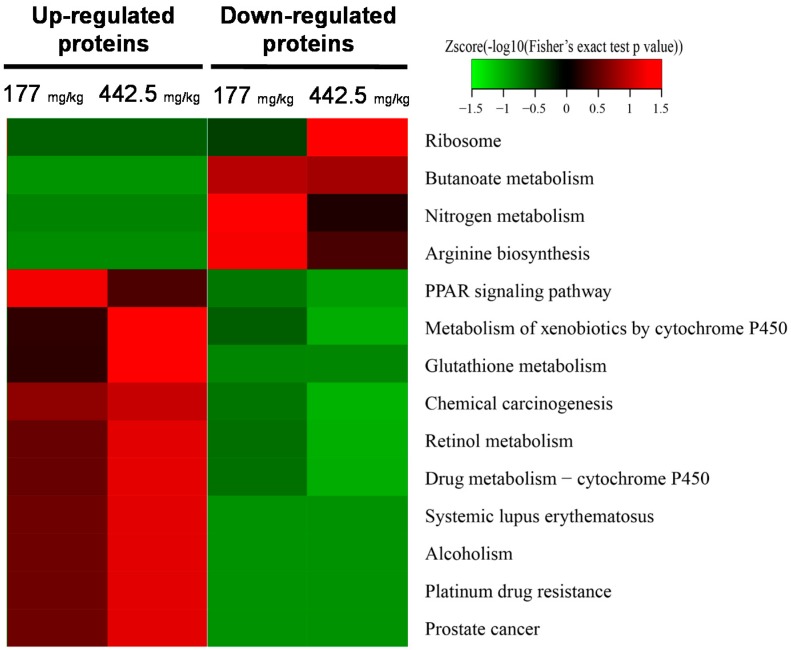
KEGG pathway enrichment-based clustering analysis of quantified proteins. The KEGG database was used to identify enriched pathways by a two-tailed Fisher’s exact test to determine the enrichment of each differentially expressed protein against all identified proteins. *p*-value < 0.05.

A total of 29 and 40 proteins were up-regulated, and 27 and 118 proteins were down-regulated after RIF administration at doses of 177 and 442.5 mg/kg, respectively (Table S2). To further characterize the mechanism of RIF-induced hepatotoxicity, we performed Gene Ontology (GO) term and Kyoto Encyclopedia of Genes and Genomes (KEGG) pathway enrichment analyses to identify the biological processes, cellular components, and molecular functions that were enriched in RIF-treated liver samples (Figure 3 and Figure 4, Figures S2 and S3). With respect to molecular function, glutathione transferase and protein dimerization activities were up-regulated in a dose-dependent manner, and steroid hydroxylase, arachidonic acid epoxygenase, and monooxygenase activities were down-regulated in a dose-dependent manner (Figure 3). In the biological process category, up-regulated proteins were involved in chromosome and chromatin organization and DNA conformational changes (Figure S2A). In addition, nuclear and chromosome-related proteins were up-regulated and cytosolic and ribosomal proteins were down-regulated in RIF-treated livers (Figure S2B).

Based on the KEGG pathway enrichment-based clustering analysis, the peroxisome proliferator-activated receptor-γ (PPARγ) signaling pathway, metabolism of xenobiotics by CYP, glutathione metabolism, chemical carcinogenesis, retinol metabolism, drug metabolism, and alcoholism-related proteins increased in a dose-dependent manner in RIF-treated livers (Figure S3). The up-regulated proteins list is shown in Table S3. In particular, significant protein enrichment was observed in the KEGG reference pathways, as shown in Figure 4. The PPARγ signaling pathway was enriched in the up-regulated group, including apolipoprotein C-III, acyl-CoA-binding protein, 3-ketoacyl-CoA thiolase A and B, and perilipin-2, which increased, but not dose-dependently. Pathways for drug metabolism, the glutathione pathway, and alcoholism were enriched for up-regulated genes and these increases were dose-dependent. The up-regulated proteins after RIF administration were CYP2A5, 2B10, 2C55, and 3A11 in the metabolism pathway and glutathione *S*-transferases (GST) Mu 1, 2, and 3, A1, and theta 2 in the GST activity pathway. GST are important phase II metabolic enzymes to protect the cells from oxidative stress and a super-gene family with detoxification function. Recent studies found that the content of GST was increased in diseased liver tissues and serums [18]. In addition, two pathways involved in systemic lupus erythematosus and alcoholism included the histones H2A, H2B, H3, and H4.

## 3. Discussion

Anti-tuberculosis drugs are a leading cause of DILI, explaining 58% of all cases of DILI and 5–22% of drug-induced acute liver failure cases [19]. RIF is the most widely used first-line antituberculosis drug, but has well-known hepatotoxicity [15]. Previous studies have shown that RIF-induced liver injury is related to oxidative stress in mitochondria, apoptotic liver cell injury in rodents, cholestasis, and hepatic lipid accumulation [15,20]. In the present study, the effects of RIF on protein profiles were examined after oral administration at doses of 177 and 442.5 mg/kg for 14 days using global proteomic technology to predict RIF-mediated liver injury.

We evaluated liver injury in mice after RIF administration by traditional clinical chemistry and histological analyses (Figure 1). Increased serum ALT/AST was an indicator of hepatotoxicity, and no change in ALP levels indicated a type of mixed liver injury via hepatocellular and cholestatic injury based on the clinical presentation [21]. Here, we explored the effects of RIF on the liver and the underlying molecular mechanisms using global proteomic technology. RIF induces CYP3A via the activation and overexpression of PXR [16]. PXR induces drug-metabolizing enzymes, e.g., CYP2B6, 2C9, 2C19, and 3A4, and transporters, such as the ATP-binding cassette transport ABCB1, following regulation at the transcriptional level. In addition, PXR is related to the homeostasis of endogenous chemicals, such as bile acids, bilirubin steroid hormones, glucose, and lipids [22]. Our results showed that CYP2C-catalyzed omeprazole 5-hydroxylation and CYP3A-mediated midazolam hydroxylation increased in response to RIF in a dose-dependent manner (Figure S1). The increased activity levels of CYP2C and 3A were correlated with the up-regulation of CYP2A5, 2B10, 2C55, and 3A11 at 442.5 mg/kg RIF (Tables S4 and S5). The increases in the activity and expression levels of these proteins were highly consistent with PXR activation by RIF.

RIF-induced hepatic lipid accumulation is associated with up-regulated PPARγ in the mouse liver according to a recent study [15]. When mice were orally administered RIF (200 mg/kg) daily for up to four weeks, hepatic lipids accumulated by the up-regulation of PPARγ via the activation of hepatic PXR. In the KEGG pathway enrichment analysis, the PPARγ signaling pathway also was enriched after RIF administration; however, it did not show dose-dependence. When mice were administered 177 mg/kg RIF, proteins in the PPARγ signaling pathway were more enriched than they were after 442.5 mg/kg RIF administration. There are different toxic responses following the administration of low and high doses of RIF. Lipid accumulation is a major toxic effect of RIF at the level of LD_10_ (177 mg/kg), while another mechanism is more important at the level of LD_25_ (442.5 mg/kg) than lipid accumulation.

We detected five proteins downstream of PPARγ in the up-regulated PPARγ signaling pathway after RIF treatment in mice, i.e., apolipoprotein C-III, acyl-CoA-binding protein, 3-ketoacyl-CoA thiolase A and B, and perilipin-2 (Tables S4 and S5). Among them, perilipin is a PAT family protein controlled by PPARγ; it coats lipid droplets in adipocytes with a phospholipid monolayer and plays a significant role in lipid metabolism, including the maturation and metabolism of lipid droplets [23,24]. Perilipin is known to suppress lipolysis, a process that breaks down triglycerides into glycerol and free fatty acids for use in metabolism [25,26]. A previous study showed that the expression of perilipin tends to increase depending on the hepatocyte lipid content of the fatty liver in humans and that the expression of perilipin affects the maturation of lipid droplets in hepatocytes [27,28]. 

In human, the RIF-induced liver injury has been suggested due to RIF-induced oxidative stress and elevated toxic metabolites caused by CYP induction [29,30]. In the present study, we suggested the mechanism of lipid accumulation by RIF-treatment was involved in the up-regulated PPARγ signaling pathway.

Among the down-regulated CYPs, the expression and activation of CYP1A2 is detected in response to drug metabolism, and CYP1A2 down-regulation is involved in fatty liver formation [31]. According to the previous study, a 44% decrease in the activation of CYP1A2 was detected in the liver cells of a fatty liver disease patient compared with the controls [32]. Therefore, the down-regulation of CYP1A2 after RIF administration in the mouse model in this study might be related to fatty liver disease. Increased plasma cholesterol, indicating fatty liver, after RIF treatment is related to decreased CYP1A2 in RIF-treated mouse livers.

Based on the KEGG pathway enrichment-based clustering analysis, CYP3A11 was up-regulated in response to RIF administration, while CYP1A2 and CYP2E1 were down-regulated (Tables S4 and S5). In humans, CYP3A4 is a major enzyme involved in drug metabolism and is frequently found in the liver [33]. CYP3A is related to drug metabolic processes and the synthesis of cholesterol, steroid, and lipid components. CYP3A4 has epoxygenase activity and metabolizes arachidonic acid into epoxyeicosatrienoic acid; it also has fatty acid monooxygenase activity and metabolizes arachidonic acid into 20-hydroxyeicosatetranoic acid (20-HETE) [34,35]. 20-HETE is known to stimulate the growth of various cancers, including breast cancer. In addition, 20-HETE is elevated in the urine of cirrhosis patients and may have harmful effects on liver health [36].

Both 4β-hydroxycholesterol (4βHC) and 6β-hydroxycortisol (6βHCL) are sensitive endogenous biomarkers for the assessment of in vivo CYP3A activity [37]. 4βHC and 6βHCL are induced following RIF administration; a daily dose of 500 mg of RIF causes a four-fold increase in 4β-OHC [38,39]. In this study, the up-regulated total cholesterol and LDL-C in response to RIF treatment might be a result of high expression of CYP3A. Bilirubin is a natural and potent antioxidant that accumulates in the blood of newborn children and leads to physiological jaundice; its clearance requires the expression of uridine 5′-diphospho-glucuronosyltransferase (UGT) [40]. In this study, PXR was identified as both a positive and negative regulator of the *UGT1A1* gene, which PXR represses during development [41]. Hyperbilirubinemia after RIF treatment might be caused by a negative regulatory effect of PXR on UGT1A expression. Based on KEGG and GO enrichment analyses, glutathione metabolism and GST activity were up-regulated by RIF (Figure 2 and Figure 3). GSTs, a major family of detoxification/cytoprotective enzymes, occur ubiquitously in the body, and the induction of GSTs, as cytoprotective phase II enzymes, is a principal strategy in the deactivation of potential carcinogens [42]. GSTs are up-regulated as a compensatory mechanism after the depletion of GSH and increased oxidative stress by RIF [29].

## 4. Materials and Methods

### 4.1. Experimental Design and Animal Treatment

Pathogen-free male ICR mice were purchased from Orient Bio Inc. (Seongnam, Korea). Upon receipt, mice were randomly divided into groups of four to five in cages and were acclimated for one week in strictly controlled conditions at 23 ± 3 °C and 50 ± 10% relative humidity, with a 150–300 lux light source on a 12 h light/dark cycle. All animal procedures followed the guidelines recommended by the Society of Toxicology (Reston, VA, USA) in 1989 and were approved by the Institutional Review Board at Kyungpook National University (approval No. 2016-57-2, approval date 28 June 2016).

The 50% lethal dose (LD_50_) of RIF is 885 mg/kg in mice; accordingly, RIF (Yuhan Co., Seoul, Korea) was administered orally to animals at 0, 177 (LD_10_), and 442.5 mg/kg(LD_25_) once a day for two weeks. RIFwas administered in the form of a mixture of solvents consisting of 5%(*v*/*v*) DMSO and 25%(*v*/*v*) polyethylene glycol (PEG) in water [43].

### 4.2. Hepatotoxic Parameters and Histology

After 14 days of administration, mice were sacrificed to obtain blood and liver samples. Prior to necropsy, all animals were fasted overnight for approximately 18 h. The animals were anesthetized with isoflurane (Ifran liquid, Hana Pharm, Co., Ltd., Seoul, Korea) and blood samples of approximately 1 mL were collected from the abdominal aorta. The blood was placed in evacuated tubes containing K2-EDTA as anticoagulant (BDTM Vacutainer, Becton Dickinson, Franklin Lakes, NJ, USA) and used for the plasma biochemical tests.

To obtain histology slides of the liver, the entire liver was weighed and two 1-cm liver samples were taken from the two major lobes. They were put in vials with Formalin Solution, neutral buffered, 10% (Sigma Aldrich, St. Louis, MO, USA), and fixed at room temperature. Histology slides were produced at Histoire (Ansan, Korea). Slides were stained with H&E and observed at 100× and 200× magnification.

### 4.3. Liver Sample Preparation for Proteomic Analysis

For proteomic profiling, the mouse livers were added to four volumes (*w*/*v*) of 0.1 M phosphate buffer (pH 7.4) with 10 mM phenylmethylsulfonylfluoride (PMSF) and manually homogenized using a glass homogenizer (Wheaton, #357538, Millville, NJ, USA) until completely broken. Liver homogenates were stored at −80 °C for the prevention of protein degradation until they were used. A Bradford assay (BioRadLaboratories, Hercules, CA, USA) was performed to measure the protein concentration of homogenates.

In total, 100 µg of protein samples were pooled with the same amount for biologically replication, according to Figure 1. For protein precipitation, the liver homogenates (200 µg of protein in 90 µL of 0.1 M phosphate buffer) were added 10 µL of trichloroacetic Acid (TCA) and incubated for 4 h at 4 °C. The precipitated samples were centrifuged 12,000× *g* for 10 min at 4 °C and withdraw the supernatant. The protein pellets were washed by 500 µL of ice-cold acetone and centrifuged again as described twice. Finally, protein pellets were dried by speed-vacuum (Lebconco, Kansas City, MO, USA) for 1 min to remove acetone and suspended in 100 mM triethyl ammonium bicarbonate (TEAB) to obtain a final volume of 100 μL. Then, 5 μL of 200 mM trisphosphine hydrochloride was added to each sample and incubated at 55 °C for 1 h for reduction. Next, 5 μL of 375 mM iodoacetamide was added to the sample, followed by incubation at room temperature for another 30 min with light protection. After incubation, the samples were mixed with pre-chilled (−20 °C) acetone and frozen at −20 °C until the formation of a precipitate. For the digestion of the acetone-precipitated protein pellet, the pellet was resuspended in 100 μL of TEAB, and re-measured protein amount using Bradford assay. After reduction/alkyation, 50 μg of proteins were transferred at new tubes and 2.5 μg of trypsin was added to each sample for overnight digestion at 37 °C.

According to procedure, 0.8 mg of 6-plex-TMT Label Reagent (Pierce Biotechnology, Rockford, IL, USA) in 41 μL of acetonitrile was added to trypsin-digested peptides for the chemical tagging of samples (vehicle control, TMT-126 and 129; low dose RIF, TMT-127 and 130; High dose RIF, TMT-128 and 131), and the reaction was processed for 1 h at room temperature. The reaction was stopped by adding 8 μL of 5% hydroxylamine. Each sample was collected and mixed in a new tube and fractionated using the Pierce™ High pH Reversed-Phase Peptide Fractionation Kit (Pierce Biotechnology). After elution, samples were dried by vacuum centrifugation and then desalted by C18 Ziptip (Millipore, Darmstadt, Germany) according to procedure. The desalted samples were dried by vacuum centrifugation and resuspended in 25 μL of 0.1% formic acid solution before they were analyzed by LC-MS/MS.

### 4.4. NanoLC-Mass Spectrometry

Chromatographic separation was performed using a custom-made capillary column (10 cm length, 75 μm internal diameter) packed with Jupiter C12 resin (4 μm particle size, 90 Å pore size; Phenomenex Inc., Torrance, CA, USA). The NanoLC-1D Plus HPLC System (Eksigent Technologies LLC, Dublin, CA, USA) was used for gradient elution at a constant flow of 300 nL/min. The LC solventswere as follows: A (water, 0.1% formic acid) and B (acetonitrile, 0.1% formic acid). The mobile phase was programmed as follows for gradient elution: (minutes, %B) = (0, 5); (12, 5); (40, 10); (50, 20); (74, 90); and (76, 90). The MS/MS analysis was performed using a LTQ Orbitrap Velos Mass Spectrometer (Thermo, Waltham, MA, USA) equipped with a nanospray ion source operating in positive ion mode with the following settings: nebulizer gas at 0 (arbitrary units), curtain gas at 0 (arbitrary units), auxiliary gas at 0, ion spray voltage 1.8 kV, capillary temperature 300 °C, collision energy at 40 with HCD mode. MS data were recorded from *m*/*z* 100 to 2000 with an accumulation time of 1 s and a pause between mass ranges of 0.5 milliseconds, operating in positive mode and MS resolution was set at 70,000. MS/MS was operated using a top-15 data-dependent method with 7500 of MS/MS resolution. For all experiments, the dynamic exclusion time was set to 5 s.

### 4.5. Protein Identification and Quantification

Peptides and proteins were identified using MaxQuant 1.5 [44] with a precursor mass error of 10 ppm, monoisotopic mass selected, and a fragment ion mass error of 20 ppm. The enzyme was digested with trypsin allowing two missed cleavages. Cysteine carbamidomethylation (57.021 Da) and TMT-3plex on peptide *N*-term and lysine (229.163 Da) was searched as a fixed modification. Methionine oxidation (15.995 Da) and protein *N*-term acetylation (42.011 Da) were searched as a variable modification. A decoy search was performed, and peptides were filtered using a false discovery rate (FDR) of 0.01 and remove the reverse and potential contaminant proteins from total identified proteins. Positive identification of proteins required a minimum of two unique peptides.

For quantification of proteins by TMT labeling, the reporter ions are calculated by TMT-3plex method at MaxQaunt. All the other parameters in MaxQuant were set to default values. In addition, quantified proteins were defined that up to 1.5 ratio is up-regulated proteins and ratio of below 0.666 is down-regulated proteins.

The mass spectrometry proteomics data have been deposited to the ProteomeXchange Consortium via the PRIDE [45] partner repository with the dataset identifier PXD006313.

### 4.6. Bioinformatics

The GO annotation proteome was obtained from the UniProt GO database (http://www.ebi.ac.uk/GOA/). The identified protein ID was converted to the UniProt ID and mapped to GO IDs. If identified proteins were not annotated in the UniProt GOA database, InterProScan was used to annotate GO functions using a protein sequence alignment method. Then, proteins were classified based on GO annotation into three categories: biological process; cellular component; and molecular function. For KEGG annotation, the KEGG database was used to annotate protein pathways. First, the KEGG online service tool KAAS was used to annotate proteins based on KEGG database descriptions. Then, the annotation results were mapped to the KEGG pathway database using the KEGG online service tool KEGG mapper.

For each category, a two-tailed Fisher’s exact test was employed to test the enrichment of each differentially expressed protein against all identified proteins. Correction for multiple hypothesis testing was performed using standard false discovery rate control methods. GO results with a corrected *p*-value of <0.05 were considered significant. The KEGG database was used to identify enriched pathways by a two-tailed Fisher’s exact test to determine the enrichment of each differentially expressed protein against all identified proteins. Correction for multiple hypothesis testing was performed using standard false discovery rate control methods. Pathways with a corrected *p*-value of <0.05 were considered significant. These pathways were classified into hierarchical categories according to the KEGG website.

### 4.7. Statistics

The hepatotoxic parameters were analyzed using Statistical Package for Social Sciences software (SPSS 22.0 K, IBM, Seoul, Korea), and all values are presented as mean ± standard error (S.E). All statistical analyses were performed with an Independent t-test that was performed to compare the results at the termination of the experiment. Statistical significance was set at *p <* 0.05 and *p <* 0.01.

## 5. Conclusions

The present study clarified the molecular signature of RIF-induced liver injury by a comparative proteomics approach. We identified a total of 1101 proteins and quantified 1038 proteins. Twenty-nine and 40 proteins were up-regulated and 27 and 118 proteins were down-regulated in response to 177 and 442.5 mg/kg RIF. In GO and KEGG pathway enrichment analysis, the PPARγ signaling pathway and cytochrome P450 were deeply linked to RIF-induced liver injury. Taken together the approach can be applied to predict DILI in non-clinical trial prior to the release of new drugs.

## Figures and Tables

**Table 1:** Effect of rifampicin on body and liver weights in male ICR mice.

Dose (mg/kg)	Body Weight (g)			Liver Weight (g)	Weight Ratio (Liver/Body Weight)
	0 Day	7 Days	14 Days	14 Days	
0	26.4 ± .2	30.1 ± 2.1	33.1 ± 2.8	1.9 ± 0.23	5.8 ± 0.46
177	26.3 ± 0.9	30.6 ± 1.5	34.5 ± 2.4	3.2 ± 0.62 *	9.1 ± 1.1 *
442.5	26.3 ± 1.0	30.8 ± 1.6	34.2 ± 2.5	4.5 ± 0.74 **	12.7 ± 1.4 **

Male ICR mice were orally treated with 177 and 442.5 mg/kg rifampicin in 5% (*v*/*v*) dimethyl sulfoxide and 25% (*v*/*v*) polyethylene glycol (PEG) in water for 14 consecutive days. All animals were subjected to necropsy 24 h after treatment. Values represent means ± S.E. of 4 animals. The asterisks indicate the significant differences in comparisons with the vehicle control at *p* < 0.05 (*) and *p* < 0.01(**).

**Table 2:** Plasma biochemical parameters after rifampicin treatment for 14 consecutive days in male mice.

Dose (mg/kg)	ALT (IU/L)	AST (IU/L)	ALP (KA Units)	Total Bilirubin (mg/dL)	Total Triglycerides (mg/dL)	Total Cholesterol (mg/dL)
0	85.5 ± 3.1	140.5 ± 3.6	24.2 ± 2.1	0.2 ± 0.1	43.7 ± 2.7	65.5 ± 5.8
177	185.2 ± 39.2 *	244.3 ± 28.6 *	29.4 ± 2.2	0.9 ± 0.5	31.8 ± 2.7	153.2 ± 29.4 *
442.5	289.3 ± 55.3 *	406.2 ± 80.6 *	28.6 ± 7.2	1.8 ± 0.6	24.1 ± 2.6	224.7 ± 48.0 *

Male ICR mice were orally treated with 177 and 442.5 mg/kg rifampicin in 5% (*v*/*v*) DMSO and 25% (*v*/*v*) polyethylene glycol (PEG) in water for 14 consecutive days. All animals were subjected to necropsy 24 h after treatment. Values represent means ± S.E. of 4 animals. The asterisks indicate significant differences in comparison with the vehicle control at *p* < 0.05 (*). AST; aspartate transaminase, ALT; alanine aminotransferase, and ALP; alkaline phosphatase. Values for AST, ALT, and ALP indicate their enzyme activities in plasma. Values for total bilirubin, triglyceride and cholesterol are their concentrations in plasma.

## Supplementary Materials

Supplementary materials can be found at www.mdpi.com/1422-0067/18/7/1417/s1.
