# Supplementary material for: Mechanism Investigation of Rifampicin-Induced Liver Injury Using Comparative Toxicoproteomics in Mice
Source: Int J Mol Sci. 2017 Jul 2;18(7):1417. doi: 10.3390/ijms18071417 (PMC5535909; doi:10.3390/ijms18071417)
Supplement: Supplementary file 1 [file ijms-18-01417-s001.zip › IJMS_DILI_RIF_revised suppl.pdf]

Supplementary material

Mechanism investigation of rifampicin-induced liver injury using comparative toxicoproteomicsin mice

Ju-Hyun Kim, Woongshik Nam, SunJoo Kim, Oh Kwang Kwon, Eun Ji Sung, Jung Jae Jo, Riya Shresha, Tae Hee Lee, Tae Won Jeon, Sung Hwan Ki, Hye Suk Lee, and Sangkyu Lee

Supplemental Methods

Determine of plasma biochemical parameters in mouse plasma

The following parameters were analyzed using an automatic analyzer (7020, Hitachi, Japan): alanine aminotransferase (ALT), aspartate aminotransferase (AST), total bilirubin, triglyceride, total cholesterol, lactate dehydrogenase (LDH), high-density lipoprotein cholesterol (HDL-C), low-density lipoprotein cholesterol (LDL-C), creatine phosphokinase (CPK), glucose, blood urea nitrogen (BUN) and albumin. The activity of alkaline phosphatase (ALP) was determined according to an instruction manual prepared by the manufacturer (Asan Pharm. Co., Hwasung, Korea).

CYP activity screening

A CYP probe assay was performed to measure liver microsome activity in RIF-treated mice. The liver homogenate S9 fraction, 0.1 M potassium phosphate buffer (pH 7.4), CYP probe substrates, and beta-NADPH-generation system (NGS) were mixed and incubated for 1 hour in a total volume of 100 µL. NGS was composed of 0.1 M glucose-6-phosphate, 10 mg/mL β-NADPH, and 1.0 U/mL glucose-6-phosphate dehydrogenase. The S9 fraction was prepared by centrifugation of the mouse homogenate at 9,000×*g* for 20 minutes. CYP probe substrates were composed of 20 µM omeprazole for CYP2C and 2.5 µM midazolam for CYP3A. After incubation, 100 µL of 0.1% formic acid in acetonitrile was added to terminate the reaction. After mixing, centrifugation was performed at 13,000×*g* for 10 minutes, and a 10-µL aliquot was added to the vial for the LC-MS/MS analysis.

Table S1. Plasma biochemical parameters after 14 consecutive days of rifampicin administration in male mice

Dose (mg/kg)	LDH (IU/L)	HDL-C (mg/dL)	LDL-C (mg/dL)	CPK (IU/L)	Glucose (mg/dL)	BUN (mg/dL)	Albumin (mg/dL)
**0**	823.8 ± 67.8	13.1 ± 0.6	10.5 ± 0.7	468.1 ± 44.7	92.4 ± 3.9	23.1 ± 0.3	3.1 ± 0.1
**177**	1,626.6 ± 312.6*	20.4 ± 2.8	46.7± 5.9	880.1 ± 83.2*	107.6 ± 9.6	23.2 ± 0.2	2.9 ± 0.1
**442.5**	2,557.4 ± 464.1*	19.4 ± 1.0	54.7 ± 7.2	1,165.9 ± 206.5*	122.3 ± 5.3	23.7 ± 0.2	2.7 ± 0.3

Male ICR mice were orally treated with 177 and 442.5 mg/kg rifampicin in 5% (v/v) DMSO and 25% (v/v) polyethylene glycol (PEG) in water for 14 consecutive days. All animals were subjected to necropsy 24 h after treatment. Value represent means ± SE of 4–6 animals. The asterisks indicate significant differences in comparisons with the vehicle control at P<0.05. LDH; activity of lactate dehydrogenase, HDL-C;content of high-density lipoprotein cholesterol, LDL-C; content of low-density lipoprotein cholesterol, CPK; activity of creatine phosphokinase and BUN; content of blood urea nitrogen. The asterisks indicate the significant differences in comparisons with the vehicle control at P<0.05 (*).

Table S2. Summary of differentially expressed proteins

Dose	Up-regulated (>1.5)	Down-regulated (<0.667)
177 mg/kg *vs.* VH	29	27
442.5 mg/kg *vs.* VH	40	118

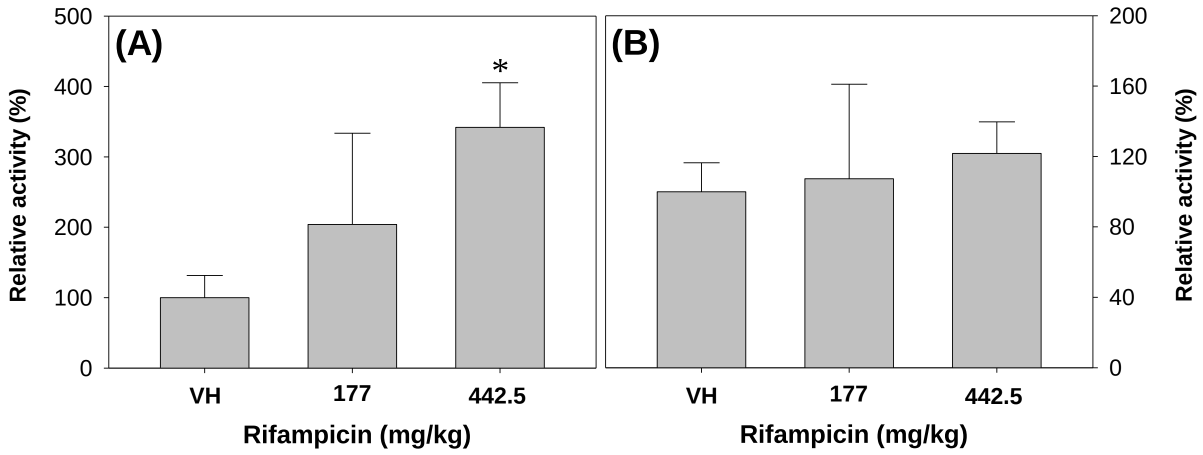

Figure S1. Enzyme activity levels of CYP2C-mediated omeprazole 5-hydroxylase (A) and CYP3A-catalyzed midazolam hydroxylase (B). The asterisks indicate the significant differences in comparisons with the vehicle control at $P<0.05$ (*).

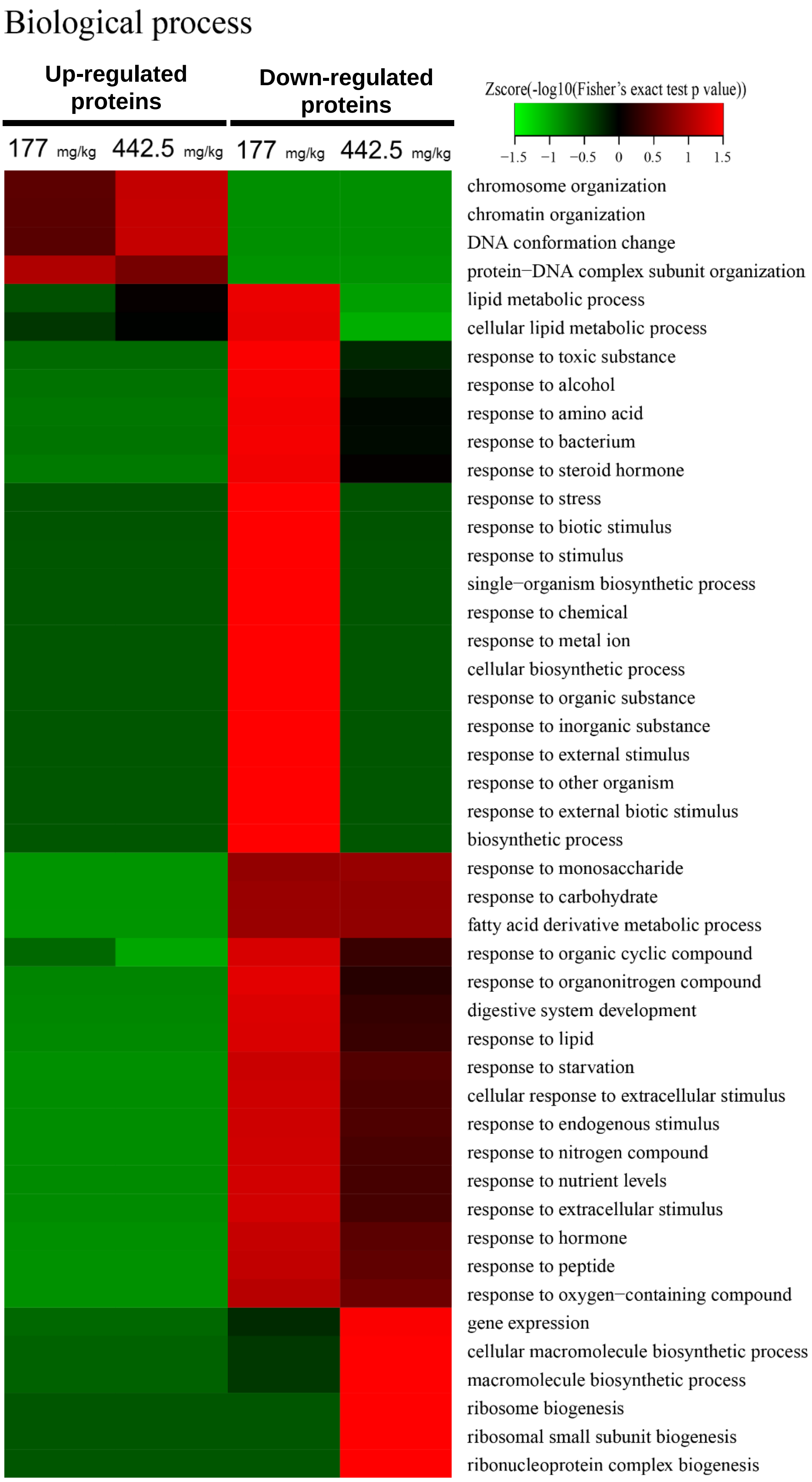
Biological process
Up-regulated proteins
Down-regulated proteins
177 mg/kg
442.5 mg/kg
177 mg/kg
442.5 mg/kg
Zscore(-log10(Fisher's exact test p value))
−1.5 −1 −0.5 0 0.5 1 1.5
chromosome organization
chromatin organization
DNA conformation change
protein−DNA complex subunit organization
lipid metabolic process
cellular lipid metabolic process
response to toxic substance
response to alcohol
response to amino acid
response to bacterium
response to steroid hormone
response to stress
response to biotic stimulus
response to stimulus
single−organism biosynthetic process
response to chemical
response to metal ion
cellular biosynthetic process
response to organic substance
response to inorganic substance
response to external stimulus
response to other organism
response to external biotic stimulus
biosynthetic process
response to monosaccharide
response to carbohydrate
fatty acid derivative metabolic process
response to organic cyclic compound
response to organonitrogen compound
digestive system development
response to lipid
response to starvation
cellular response to extracellular stimulus
response to endogenous stimulus
response to nitrogen compound
response to nutrient levels
response to extracellular stimulus
response to hormone
response to peptide
response to oxygen−containing compound
gene expression
cellular macromolecule biosynthetic process
macromolecule biosynthetic process
ribosome biogenesis
ribosomal small subunit biogenesis
ribonucleoprotein complex biogenesis

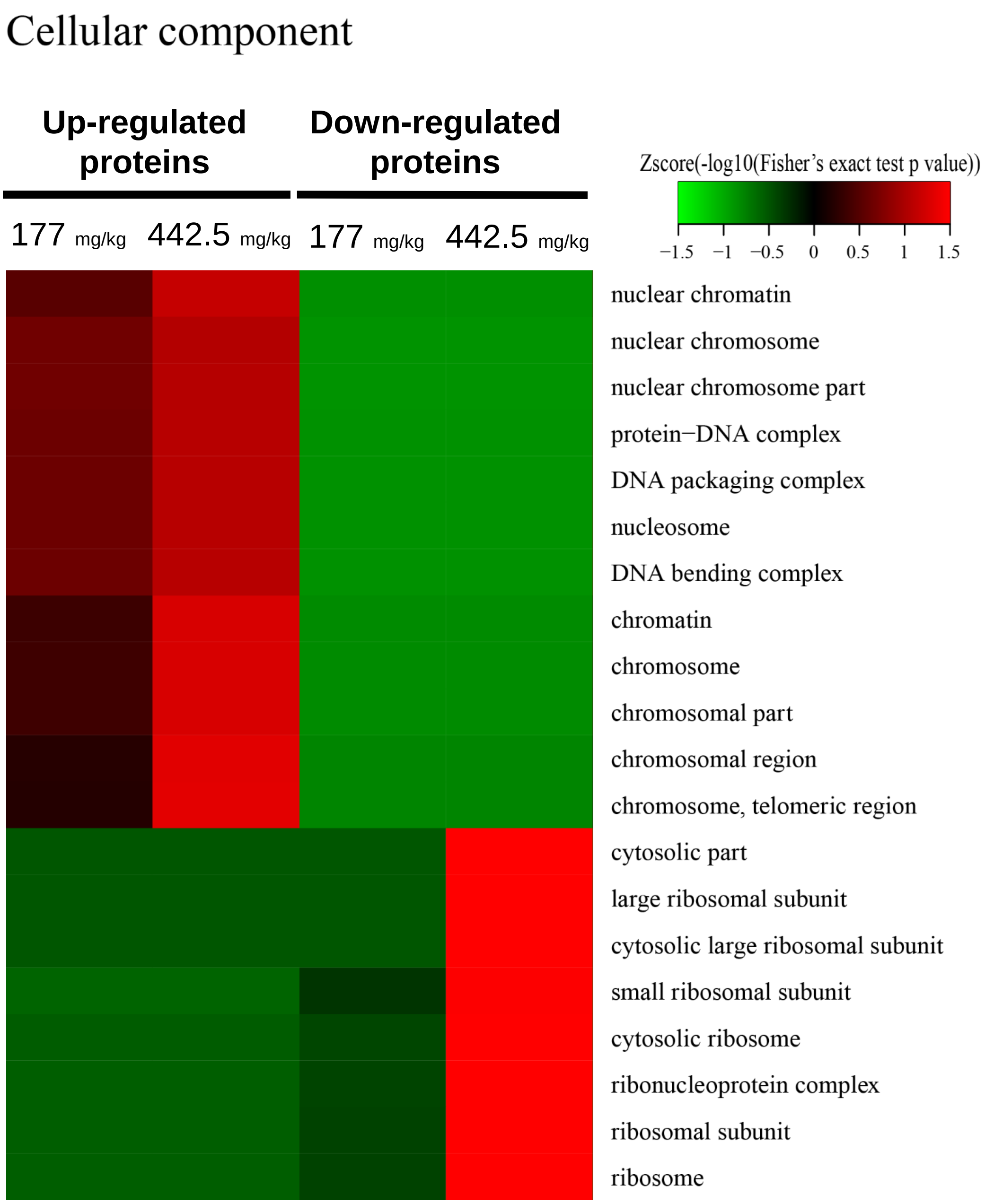

Figure S2. Gene ontology functional classification of the quantified proteins based on biological process (A), and subcellular location (B).The heatmap was generated using the two-tailed Fisher's exact test was employed to test the enrichment of each differentially expressed protein with P-value of <0.05 against all identified proteins.

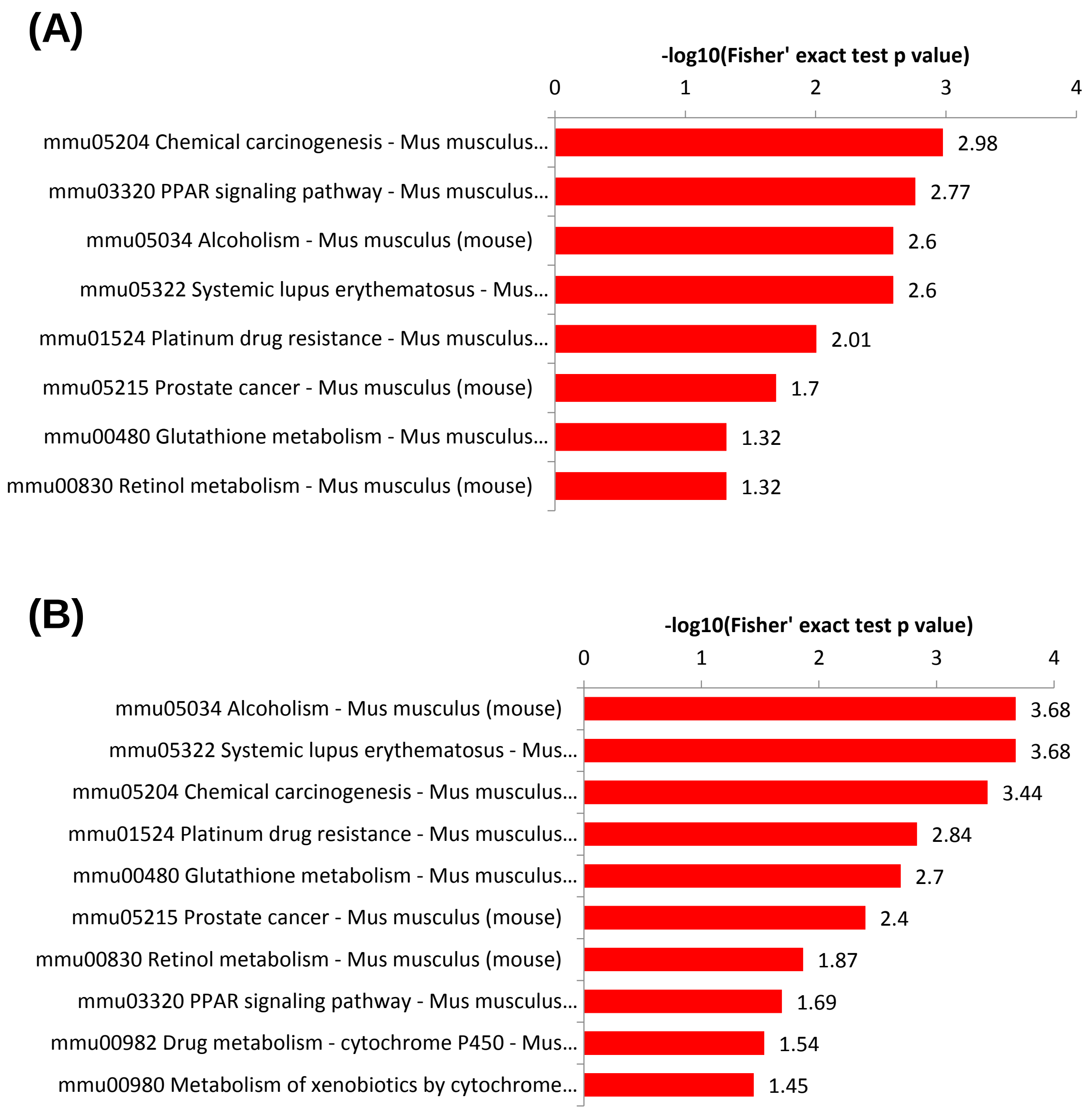

Figure S3. Enrichment analysis of KEGG pathway of up-regulated proteins after the oral administration of 177 mg/kg (A), and 442.5 mg/kg (B) rifampicin.
